# A Rare Case of *Raoultella planticola* Urinary Tract Infection in a Patient With Immunoglobulin A Nephropathy

**DOI:** 10.1177/2324709618780422

**Published:** 2018-06-05

**Authors:** Hassan Mehmood, Najwa Pervin, Muhammad Israr Ul Haq, Khushbakht Ramsha Kamal, Asghar Marwat, Muzammil Khan

**Affiliations:** 1 Temple University, Philadelphia, PA, USA; 2 Conemaugh Memorial Medical Center, Johnstown, PA, USA; 3 Southern Illinois University, Springfield, IL, USA; 4 NYU Langone Medical Center, Brooklyn, NY, USA; 5 Fatima Jinnah Medical University, Lahore, Punjab, Pakistan

**Keywords:** *Raoultella planticola*, urinary tract infection, IgA nephropathy, antibiotic

## Abstract

*Raoultella planticola* is a gram-negative, aerobic, nonmotile mostly found in environments with high prevalence in soil and water. This organism is a very rare human pathogen as only 29 cases of *Raoultella planticola*–related infections have been reported until 2017, with only 7 cases in the United States. Only 3 cases of urinary tract infection secondary to *R planticola* have been reported, 1 in a pediatric patient and 2 in adults. In this article, we present a case of *R planticola* urinary tract infection in a 65-year-old male with immunoglobulin A nephropathy. On investigation, the patient was found to be septic and empirical antibiotic was started for gram-negative coverage. The patient showed remarkable improvement and discharged on oral antibiotic for 7 days. *R planticola* rarely cause infection in humans, with overall good prognosis.

## Introduction

*Raoultella planticola* is a gram-negative, aerobic, nonmotile mostly found in environments with high prevalence in soil and water. It was first described in the 1980s as *Klebsiella planticola and Klebsiella trevisanii*.^1^ It was reclassified into a new genus in 2001 as *Raoultella planticola*.^2^ This organism is a very rare human pathogen as only 29 cases of *R planticola*–related infections have been reported until 2017, with only 7 cases in the United States. Only 3 cases of urinary tract infection secondary to *R planticola* have been reported, 1 in a pediatric patient and 2 in adults.^3,4^ In this article, we present a case of *R planticola* urinary tract infection in 65-year-old male with immunoglobulin A nephropathy.

## Case Presentation

A 65-year-old male with past medical history of hypertension, diabetes mellitus type 2, hyperlipidemia, and end-stage renal diseases secondary to biopsy-proven immunoglobulin A nephropathy came to our hospital with dysuria, dark urine, and fever going on for the last 3 days. His vitals showed a temperature of 39.3°C, blood pressure of 164/89 mm Hg, pulse of 99 beats per minute, and respiratory rate of 20 breaths per minute. The examination was unremarkable except for mild lower abdominal tenderness. Initial laboratory workup was remarkable for white blood cells (WBCs) of 10 900/µL (3100-8500/µL), neutrophil percentage of 87% (25% to 62%), absolute neutrophils of 9500/µL (1700-6300/µL), platelets of 174 000/µL (140 00-440 000/µL), sodium of 135 mmol/L (136-145 mmol/L), lactic acid of 1.1 mmol/L (0.5-2.2 mmol/L), blood urea nitrogen of 47 mg/dL (9-21 mg/dL), and creatinine of 4.5 mg/dL (0.6-1.1 mg/dL). Urine analysis showed 1+ bacteria, large leukocytes, WBC >50/high-power field (HPF), and squamous epithelial cells 0 to 5/HPF. Keeping in mind urosepsis, blood cultures were drawn from 2 peripheral sites along with urine culture, and he was started on intravenous ceftriaxone and intravenous fluid as per sepsis protocol. Nephrology was consulted and the patient got dialysis the next day secondary to end-stage renal diseases. The patient started showing improvement. On day 2, the patient was afebrile, and WBC started trending down along with resolution of dysuria. Blood cultures did not grow anything, but 3 days later urine culture grew *R planticola* sensitive to all main-line antibiotics, including ceftriaxone, ciprofloxacin, nitrofurantoin, cefazolin, gentamicin, and trimethoprim-sulfamethoxazole. The patient was subsequently discharged on the fourth day on ciprofloxacin renal dose 250 mg orally every 12 hours for 4 more days to complete 7 days of treatment. The patient was seen in clinic after 2 weeks with complete resolution of symptoms.

## Discussion

*Raoultella planticola* is an encapsulated, nonmotile, aerobic, gram-negative rod predominantly found in water and soil. Although *R planticola* is mainly an aquatic and soil bacterium, it has been clinically isolated from human sputum, stool, wound, and urine. To date, 29 cases of human infection with *R planticola* has been reported with only 3 urinary tract infections.^3,4^ *R planticola* is difficult to isolate and to identify, as it can easily be confused with other genera, especially *klebsiella.*^5^ *R planticola* rarely cause infection in healthy individuals. Malignancy, transplant recipients, dialysis-dependent patients, diabetes mellitus, and immunocompromised state also put them at high risk.^6-9^

*Raoultella planticola* has been associated with cases of pneumonia, urinary tract infection, cholangitis, conjunctivitis, peritonitis, necrotizing fasciitis, bacteremia, cellulitis, and soft tissue infection. On literature review of 29 reported cases, 3 patients died, 22 patients had full recovery, and 4 patients had an unknown outcome. Mortality is high in immunocompromised patients.^3^ The First reported human infection due to *Klebsiella trevisanii* (later reclassified as *R planticola*) was in 1986, which included bacteremia in a 69-year-old patient.^10^ In 2014, a case of *R Planticola* bacteremia in a 56-year-old female was reported following consumption of seafood salad containing squid and octopus.^5^

As there is limited data regarding this pathogen, especially in humans, the mechanism of its pathogenesis remains unclear. Immunocompromised state, proton pump inhibitor use, and chemotherapy increase the chances of infection. *R planticola* has the ability to change histidine to histamine leading to scombroid poisoning when poorly cooked sea food eaten in a large quality.^5^ Variety of human organ systems had been affected, with no predilection for a particular organ system.

Culture along with VITEK-2 (bioMerieux) automated bacterial identification system not only help in identification of *R planticola* but is also highly sensitive in differentiating between *Raoultella and Klebsiella*.

Treatment of *R planticola* urinary tract is mainly empiric antibiotic for gram-negative coverage and should be narrowed accordingly when further microbiologic information is available. Usually *R planticola* is sensitive to all main-line gram-negative covering antibiotics; however, multidrug-resistant strains of *R planticola* have been isolated from both patients and the environment.^11-13^ Our patient did not show resistance to any antibiotic.

## Conclusion

In conclusion, infection due to *R planticola* has been on the rise recently. The organism has been involved in infecting multiple organ systems. Individuals who are immunocompromised, have multiple comorbid diseases, and dialysis patients are at risk. Avoidance of contaminated water is the key to prevention. *R planticola* infection has good prognosis overall. It is prudent to be aware of this human pathogen and early initiation of antibiotics is the main treatment. As with any other human pathogen, we recommend closely monitoring its pattern of resistance.
